# Clinical effect of lymphocyte immunotherapy on patients with unexplained recurrent spontaneous abortion

**DOI:** 10.1002/iid3.474

**Published:** 2021-06-08

**Authors:** Sudong Liu, Xiaodong Gu, Ruiqiang Weng

**Affiliations:** ^1^ Research Experimental Center, Meizhou People's Hospital (Huangtang Hospital) Meizhou Hospital Affiliated to Sun Yat‐sen University Meizhou China; ^2^ Guangdong Provincial Key Laboratory of Precision Medicine and Clinical Translational Research of Hakka Population Meizhou People's Hospital (Huangtang Hospital) Meizhou Hospital Affiliated to Sun Yat‐sen University Meizhou China

**Keywords:** blocking antibody (BA), live birth rate, lymphocyte immunotherapy (LIT), unexplained recurrent spontaneous abortion (URSA)

## Abstract

**Introduction:**

Lymphocyte immunotherapy (LIT) is believed to be a viable treatment for unexplained recurrent spontaneous abortion (URSA), but its effect remains controversial. This study aims to investigate the clinical effect of LIT in patients with URSA and clarify the factors that may influence the outcome of LIT.

**Methods:**

This study included a total of 704 URSA patients, of which 444 patients accepted LIT treatment. URSA patients that did not accept LIT served as control group. Clinical characteristics were collected and analyzed between LIT and control group. The blocking antibody was tested before and after LIT. The outcome of LIT treatment was recorded. Logistic regression analysis was applied to evaluate the independent predictors of LIT success.

**Results:**

After LIT treatment, 77.9% (346/444) of USRA patients turned to BA positive, and the conversion rate elevated with increased LIT (*p* < .001). LIT significantly improved the pregnancy rate and live birth rate in USRA patients (65.3% vs. 29.6%, *p* < .001; 80.3% vs. 50.6%, *p* < .001). Multivariate regression analysis suggested that younger maternal age and positive BA were independent predictors of LIT success.

**Conclusion:**

LIT effectively induced the production of BA, and improved pregnancy rate and live birth rate in URSA patients. Our findings supported LIT as a beneficial treatment for URSA.

## Graphical abstract

Immunotherapy effectively induced the production of blocking antibody. Immunotherapy improved pregnancy rate and live birth rate in patients with unexplained recurrent spontaneous abortion

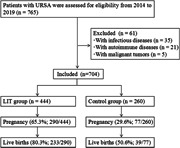

## 1 INTRODUCTION

Recurrent spontaneous abortion (RSA), which is generally defined as two or more consecutive spontaneous abortions at early pregnancy (<22 weeks) of gestation, has become a major social health problem in China.^1^ It is estimated that RSA occurs in 2%–5% of women at reproductive age and the incidence is increasing annually.^2^, ^3^ Studies show that patients with RSA are more vulnerable to miscarriage, with an odds of around 25% at third pregnancy, 45% at the fourth, and 54% at the fifth.^4^ It is urgent to find a way to reduce the incidence of RSA.

Many studies suggest that various etiologies may account for the occurrence of RSA, for example, genetic factors, endocrine causes, autoimmune diseases, infections.^5^, ^6^ However, at the present still more than 50% of RSA cases are unable to find the sources, which are termed unexplained recurrent spontaneous abortion (URSA).^7^ Previous studies have shown that more than 60% of URSA cases are caused by alloimmune mechanisms which prevent maternal immunological responses and fail to protect the semiallogeneic pregnancy.^8^, ^9^ Thus, treatments for URSA focus on immunomodulation, to induce the production of blocking antibody (BA) or cytokines. Lymphocyte immunotherapy (LIT) was first introduced by Mowbray et al.^10^ in 1985 as a treatment for URSA. However, three decades past and the effect of LIT is still controversial. A meta‐analysis of a randomized contolled trial (RCT) by Wong et al.^11^ in 2014 suggested that LIT was unable to improve the live birth rate in women with URSA. Another meta‐analysis by Liu et al.^12^ in 2016 reviewed 18 RCTs and indicated that LIT performed before and after pregnancy secured better outcomes than that performed solely before pregnancy. Recently, Chen et al.^13^ evaluated the clinical benefits of LIT in 619 URSA patients and found that LIT significantly elevated the pregnancy rate and live rate, as well as reducing the abortion rate and its effect depended on BA conversion. Many researchers believed that the effect of LIT may vary in different women.^14^ In this regard, more studies are needed to clarify the clinical factors that may be associated with benefits of immunotherapy.

This study aims to explore the clinical benefits of LIT in patients with URSA and identify clinical characteristics that influence the outcome of this treatment. The present study would add evidence to the clinical effect of LIT and provide practical advices to improve the outcome.

## 2 MATERIALS AND METHODS

### 2.1 Patients

This retrospective study included the URSA patients from January 1, 2014 to December 30, 2019 in the Reproductive Medical Center of Meizhou People's Hospital (Huangtang Hospital), Meizhou Hospital Affiliated to Sun Yat‐sen University. Eligible patients should meet the following criteria: (i) Age 18 years or above; (ii) experienced two or more early abortions (under 22 weeks); (iii) had normal reproductive tract anatomy and chromosome karyotype, negative results for antinucleic, antiphospholipid, lupus anticoagulant, and antithyroid antibodies; (iv) negative BA before LIT. Patients that had infectious diseases, autoimmune diseases, malignant tumors were excluded from our study. For patients with a history of excess thrombosis, antithrombosis treatment like aspirin and lovenox were used during LIT treatment. Some URSA patients that were unqualified for LIT treatment might receive additional treatment options such as enbrel, aspirin, lovenox, and methylprednisolone. This study was approved by the Ethics Committee of Meizhou People's Hospital (No.: MPH‐HEC 2014‐A‐01). Written informed consent was obtained from all participants. The URSA patients were divided into study group (LIT group) and control group.

### 2.2 Lymphocyte immunotherapy

Donors (patient's husband usually) were required to take blood tests to exclude infectious diseases such as AIDS. To perform LIT treatment, 30 ml of fasting venous blood was taken from donor and placed in anticoagulated tube containing EDTA. Lymphocytes were isolated by density gradient centrifugation using Ficoll‐Hypaque solution (MD Pacific Bio) following manufacturer's instruction. Finally, lymphocytes were dissolved in physiological saline at a density of 1.0 × 10^7^ cell/ml. One milliliter of lymphocyte solution was intradermally injected to the URSA patients through five points on the forearm (0.2 ml/point). LIT treatment was performed once every 3 weeks, four times as a course. Two weeks after a course, patients were tested for the production of BA. If BA turned positive, LIT treatment was performed once every 4–6 weeks, during which women were encouraged to try for a baby. However, if BA remained negative, another course of LIT was performed. After pregnancy, one treatment was consolidated for every 2–3 weeks until 16 weeks of gestation. LIT success was defined as woman in the LIT group successfully produced a live birth.

### 2.3 BA test

The BA was tested by enzyme‐linked immunosorbent assay (ELISA) kit (Lambda; provided by Beijing Suoao Biotechnology in China) following manufacturer's protocol. The ELISA kit contained immunoglobulin G antibodies against HLA class I and class II. BA positive was defined if the optical density (OD) value was above 0.2 times of positive serum control.

### 2.4 Clinical characteristics collection

Clinical characteristics of the URSA patients at the time of check‐in were collected from medical records. For the sake of privacy, patients were de‐identity before analysis. The clinical characteristics included age, body mass index (BMI), hypertension, previous miscarriages, primary RSA, hormone levels, lymphocytes levels, and MTHFR C677T polymorphisms. Other important parameters could be obtained or calculated by reviewing patients' medical records, including BA conversion, pregnancy rate, live rate, and abortion rate.

### 2.5 Statistical analysis

Statistical analyses were performed using SPSS 20.0 software (IBM Corp.). Continuous data were expressed as mean ± standard deviation (*SD*), and categorical data were expressed as number (percentage). Comparisons between two groups were tested by Student's *t* test or *χ* ^2^ test when appropriate. Binary logistic regression analysis was used to determine the variables associated with LIT success. A two‐side *p* < .05 was considered significant.

## 3 RESULTS

### 3.1 General information of study subjects

**Figure 1:**
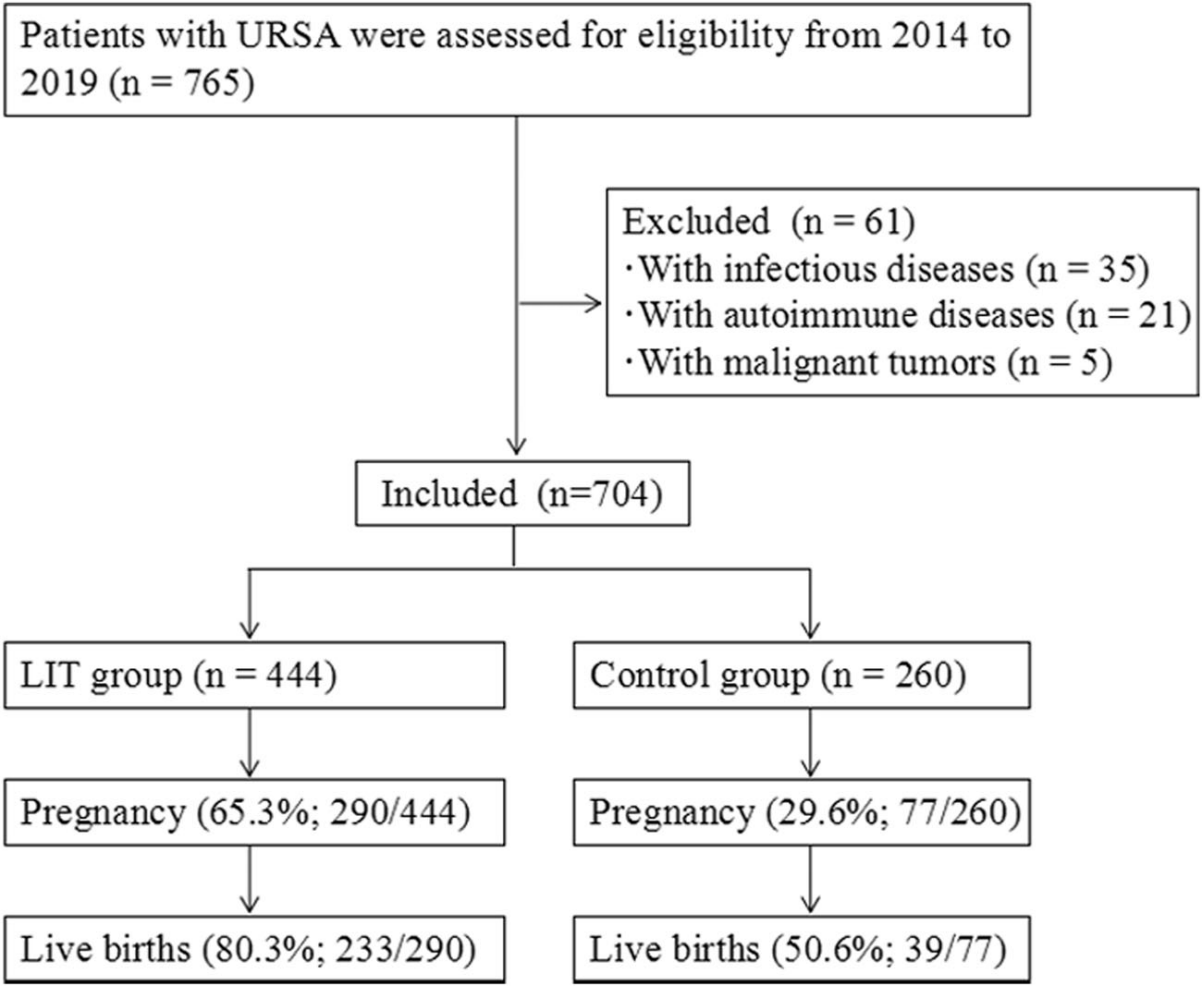
Flow chart of study population inclusion, exclusion and main findings. LIT, lymphocyte immunotherapy; URSA, unexplained recurrent spontaneous abortion

A total of 765 women were diagnosed with URSA in our hospital during this period. After excluded for infectious diseases, autoimmune diseases or malignant tumors, there were eligible 704 patients, including 444 in the LIT group and 260 in the control group (Figure 1). The general information of study subjects are presented in Table 1. The average age of URSA patients was 29.6 ± 5.1 years. These URSA patients have a mean infertility duration of 3.9 ± 0.7 years and mean miscarriages of 3.0 ± 0.5. Meanwhile, about 86.8% of these patients suffered primary RSA. The clinical characteristics between LIT group and control group were compared and no statistical difference was observed.

**Table 1:** General information of the study subjects

Variables	Total (*n* = 704)	LIT group (*n* = 444)	Control group (*n* = 260)	*p* Value
Maternal age (years)	29.6 ± 5.1	29.8 ± 5.0	29.7 ± 5.4	.574
Prepregnancy BMI (kg/m^2^)	22.7 ± 5.1	22.7 ± 4.5	22.8 ± 6.2	.936
Infertility duration (years)	3.9 ± 0.7	3.9 ± 0.6	3.9 ± 0.8	.188
Miscarrage (*n*)	86	3.0 ± 0.4	3.0 ± 0.4	.565
Diabetes mellitus, *n* (%)	35 (5.0)	23 (5.2)	12 (4.6)	.690
Hypertension, *n* (%)	17 (2.4)	12 (2.7)	5 (1.9)	.515
Primary RSA, *n* (%)	611 (86.8)	384 (86.5)	227 (87.3)	.588
Basal FSH (IU/L)	5.7 ± 1.7	5.8 ± 1.6	5.7 ± 1.9	.924
Basal LH (IU/L)	4.5 ± 1.8	4.6 ± 1.9	4.4 ± 1.7	.569
Basal estrogen (pg/ml)	43.4 ± 24.7	39.2 ± 19.1	45.5 ± 28.9	.224
Blood lymphocyte (10^3^cell/μl)				
T cell	1.47 ± 0.45	1.49 ± 0.47	1.44 ± 0.42	.148
B cell	0.28 ± 0.13	0.28 ± 0.13	0.28 ± 0.13	.843
NK cell	0.42 ± 0.21	0.42 ± 0.21	0.41 ± 0.20	.818
MTHFR C677T polymorphism^a^				
CC	148 (55.0)	88 (56.8)	60 (52.6)	.500
CT	101 (37.5)	59 (38.1)	42 (57.1)	.838
TT	20 (7.4)	8 (5.2)	12 (10.5)	.097

Abbreviations: BMI, body mass index; FSH, follicle‐stimulating hormone; LH, luteinizing hormone; LIT, lymphocyte immunotherapy; NK, nature killer; RSA, recurrent spontaneous abortion

^a^ Due to the retrospective nature, we only collected the MTHFR results of 269 patients.

### 3.2 Effect of LIT on maternal BA

After LIT treatment, 346 (77.9%) URSA patients presented positive BA. We analyzed the clinical characteristics that might influence the effect of LIT on BA conversion. As shown in Table 2, risk factors that have been reported to be associated with URSA presented no significant difference between patients with positive BA and negative BA after LIT treatment. Furthermore, we found that after LIT, positive BA rate was significantly elevated in comparison with that in the control group (77.9% vs. 6.5%, *p* < .001) and increase in LIT times significantly elevated the conversion rate of maternal BA (Table 3).

**Table 2:** Comparison of characteristics between patients with positive and negative BA after LIT

Variables	Positive BA (*n* = 346)	Negative BA (*n* = 98)	*p* Value
Maternal age (years)	29.2 ± 4.8	30.1 ± 5.4	.220
Pre‐pregnancy BMI (kg/m^2^)	23.4 ± 3.5	22.6 ± 3.5	.732
Infertility duration (years)	4.0 ± 0.6	3.9 ± 0.7	.276
Miscarriage (*n*)	3.0 ± 0.4	3.1 ± 0.5	.109
Primary RSA, *n* (%)	298 (86.1)	86 (87.8)	.677

*Note*: Data are expressed as the mean ± standard deviation or number (percentage).

Abbreviations: BA, blocking antibody; BMI, body mass index; RSA, recurrent spontaneous abortion.

**Table 3:** BA conversion rate after LIT treatment

		LIT treatment			
Variable	Control group	Total	<4 times	4–6 times	>6 times
Positive BA	11.9% (31/260)	77.9% (346/444)^a^,***	52.5% (39/74)	75.7% (139/184)^b^,***	90.6% (168/186)^c^,^d^,***

Abbreviations: BA, blocking antibody; LIT, lymphocyte immunotherapy.

^a^ Comparison between LIT group and control group.

^b^ Comparison between LIT less than four times and four–six times.

^c^ Comparison between LIT four–six times and more than six times.

^d^ Comparison between LIT less than four times and more than six times.

*** *p* < .001.

### 3.3 Effect of LIT on pregnancy outcome

As shown in Table 4, the pregnancy rate significantly increased in URSA patients who undertook LIT than those did not (65.3% vs. 29.6%, *p* < .001). Patients in LIT group had a significantly higher live birth rate (80.3% vs. 50.6%, *p* < .001), as well as a lower abortion rate in comparison with those in control group (19.7% vs. 49.4%, *p* < .001).

**Table 4:** Comparison of pregnancy outcomes between the lymphocyte immunotherapy (LIT) and control group

Variables	LIT group (*n* = 444)	Control group (*n* = 260)	*p* Value
Pregnancy, *n* (%)	290 (65.3)	77 (29.6)	<.001
Live births, *n* (%)	233 (80.3)	39 (50.6)	<.001
Abortions, *n* (%)	57 (19.7)	38 (49.4)	<.001
Preterm birth, *n* (%)	11 (3.8)	3 (3.9)	.990
Birth gestational age (weeks)	38.1 ± 1.4	37.8 ± 2.1	.309
Birth weight (g)	2954.4 ± 514.5	2861.1 ± 444.3	.345

### 3.4 Independent factors that were associated with benefits of LIT

Logistic multivariate regression analysis was used to identify predictive markers for LIT success. As shown in Table 5, younger maternal age (odds ratio [OR]: 2.50; 95% confidence interval [CI]: 1.20–5.24; *p* = .015) and positive BA (OR: 2.42; 95% CI: 1.24–4.71; *p* = .009) were independent predictors of LIT success. Other factors, such as infertility duration, prepregnancy BMI, miscarriage, and primary RSA showed no significant association with LIT success.

**Table 5:** Binary logistic regression of predictive factors associated with LIT success

	Univariate analysis		Multivariate analysis	
Variables	OR (95% CI)	*p* Value	OR (95% CI)	*p* Value
Maternal age (<35 years)	2.33 (1.14–4.78)	.021	2.50 (1.20–5.24)	.015
Infertility duration (<4 years)	0.32 (0.07–1.47)	.143	0.24 (0.05–1.15)	.074
Prepregnancy BMI (<25 kg/m^2^)	0.84 (0.43–1.64)	.609	0.94 (0.47–1.88)	.869
Miscarriage (*n* < 4)	1.15 (0.23–5.70)	.793	1.44 (0.28–7.37)	.663
Primary RSA	1.21 (0.64–2.28)	.861	0.82 (0.34–1.96)	.649
Positive BA	2.35 (1.23–4.48)	.009	2.42 (1.24–4.71)	.009

Abbreviations: BA, blocking antibody; BMI, body mass index; LIT, lymphocyte immunotherapy; LIT success, live birth; RSA, recurrent spontaneous abortion.

## 4 DISCUSSION

URSA remains one of the most suffering diseases for young women subjected to pregnancy. As the exact mechanisms are still unknown, many researchers believe that imbalanced immune factors play a key role in this disease. LIT has been used to treat URSA. However, the unconfirmed effects of this therapy restrain its widespread application. The present study analyzed the clinical benefits of LIT on patients with URSA, as well as baseline characteristics that influenced the clinical benefits. Our data suggested that LIT significantly elevated the live birth rate in patients with URSA, and younger maternal age and positive BA were independent factors associated with LIT success.

Some studies have shown that immunological dysregulation play an important role in the occurrence of URSA.^6^ Fetuses are recognized as a semiallograft by maternal immune system. BA, which is spontaneously produced in mother, functions as a normal mechanism to work against natural rejection towards fetuses, thus resulting in a successful pregnancy. On the contrary, failure in the production of BA would lead to pregnancy loss.^15^ Many researchers believe that immune therapies are useful tactics for improving live births rate in cases of recurrent miscarriage. During LIT treatment, immunization with paternal lymphocytes stimulates the maternal immune system and motivates BA production that may contribute to a successful pregnancy.^16^ In the present study, we observed that 77.9% of URSA patients became BA positive after LIT and the conversion rate increased along with more LIT. Accordingly, the pregnancy rate extensively ascended after LIT, from 29.6% to 65.3%, and live birth rate significantly grew, from 50.6% to 80.3%. These results suggested that LIT significantly improved the maternal immune balance and pregnancy outcome.

The findings observed in our study are consistent with the recent study by Chen et al.,^13^ which showed that LIT significantly improved the pregnancy outcomes in URSA patients. Another study by Pourakbari et al.^17^ also indicated that LIT effectively treated URSA patients when an appropriate dose of fresh lymphocytes was injected intradermally before and during pregnancy. However, some previous studies suggested that URSA patients did not significantly benefit from LIT. A randomized trial of LIT on 183 URSA patients by Ober et al.^18^ found that immunisation with paternal mononuclear cells did not improve pregnant ending. A meta‐analysis by Achilli et al.^5^ implied that LIT may not increase the live birth rate in URSA patients, or should be used conditionally. The conflicting findings may be partly attributed to variation in research protocols such as selection criteria, diagnostic testing and treatment methods.

Some researchers have investigated the factors that may influence the clinical benefits of LIT.^2^, ^19^ Older maternal age was reported to be associated with worse outcome in patients with URSA and impaired the effect of LIT.^20^ Daya and Gunby^21^ found that URSA patients with more previous miscarriages had a lower chance of successful pregnancy. Studies suggested that production of serum antibodies, that is, anti‐idiotypic antibodies, antipaternal cytotoxic antibodies, blocking antibodies, may contribute to a successful pregnancy.^22^ The present study suggested that positive BA and younger maternal age (under 35 years) were independent predictive factors of LIT success. To be noted, our data showed that LIT was successful in younger women who were selected based on absent autoimmune factors. However, older women with URSA often suffer autoimmune factors, additional treatments such as intravenous immunoglobulin, immune modulators, and anticoagulants may considered to be used in combination with LIT treatment to gain maximum benefit.^23^, ^24^ There are other alloimmune mechanisms that have been reported to be involved in recurrent miscarriage, for example, natural killer cells (NK) hyperactivity, T‐helper 1 (Th1), and Th2 imbalance.^25^, ^26^ The present study compared the absolute counts of blood lymphocytes (T/B/NK cell) in LIT group and control group, but no significant difference was observed.

There are some limitations in this study. First, the retrospective nature of the study made it difficult to avoid selection bias and confirm the accuracy of clinical data. Second, some immune markers of RSA, such as Th17/Treg cell ratios, Th1/Th2 cytokine ratios have not been assessed. Third, some URSA patients who did not get pregnant within 3–6 months after LIT may choose to undergo in vitro fertilization (IVF). The present study did not investigate the effect of LIT on IVF.

## 5 CONCLUSIONS

The present study suggested that LIT effectively induced the production of BA and improved pregnancy rate and live birth rate in patients with URSA. Furthermore, BA conversion and younger maternal age were independent factors that were associated with the effects of LIT. These findings supported LIT as a beneficial treatment for patients with URSA.

## CONFLICT OF INTERESTS

The authors declare that there are no conflict of interests.

## AUTHOR CONTRIBUTIONS

*Project development and manuscript editing*: Sudong Liu. *Data analysis and manuscript writing*: Xiaodong Gu. *Data collection*: Ruiqiang Weng.

## ETHICS APPROVAL

This study was approved by the Ethics Committee of Meizhou People's Hospital (No. MPH‐HEC 2014‐A‐01).

## Data Availability

The datasets used and analyzed during the current study will be provided by the corresponding author upon reasonable request.
